# Inhibition of Sirtuin 3 prevents titanium particle-induced bone resorption and osteoclastsogenesis via suppressing ERK and JNK signaling

**DOI:** 10.7150/ijbs.53992

**Published:** 2021-04-03

**Authors:** Ning Li, Xiaoping Li, Kai Zheng, Jiaxiang Bai, Weicheng Zhang, Houyi Sun, Gaoran Ge, Wei Wang, Zhen Wang, Ye Gu, Yi Xue, Yaozeng Xu, Dechun Geng, Jun Zhou

**Affiliations:** 1 Department of Orthopaedics, The First Affiliated Hospital of Soochow University, No. 188 Shizi Street, Suzhou, Jiangsu 215006, China.; 2 Department of Orthopaedics, Suzhou Kowloon Hospital Shanghai Jiao Tong University School of Medicine, Suzhou, Jiangsu 215006, China.; 3 Department of Orthopedics, Soochow University Affiliated First People's, Hospital of Changshou City, Changshu, Jiangsu 215500, China.; 4 Department of Orthopaedics, Changshu Hospital Affiliated to Nanjing University of Traditional Chinese Medicine, Suzhou, Jiangsu 215500, China.

**Keywords:** Sirtuin 3, peri-prosthetic osteolysis, osteoclasts, inflammatory factors

## Abstract

Implant-derived wear particles can be phagocytosed by local macrophages, triggering an inflammatory cascade that can drive the activation and recruitment of osteoclasts, thereby inducing peri-prosthetic osteolysis. Efforts to suppress pro-inflammatory cytokine release and osteoclastsogenesis thus represent primary approaches to treating and preventing such osteolysis. Sirtuin 3 (SIRT3) is a NAD^+^-dependent deacetylases that control diverse metabolic processes. However, whether SIRT3 could mitigate wear debris-induced osteolysis has not been reported. Herein we explored the impact of the SIRT3 on titanium particle-induced osteolysis. Tartrate resistant acid phosphatase (TRAP) staining revealed that the inhibition of SIRT3 suppressed nuclear factor-κB ligand (RANKL)-mediated osteoclasts activation in a dose-dependent fashion. Notably, inhibition of SIRT3 also suppressed matrix metallopeptidase 9 (MMP9) and nuclear factor of activated T‐cell cytoplasmic 1 (NFATc1) expression at the mRNA and protein levels, while also inhibiting the mRNA expression of dendritic cell-specific transmembrane protein (DC-STAMP), ATPase H^+^ Transporting V0 Subunit D2 (Atp6v0d2), TRAP and Cathepsin K (CTSK) . In addition, inhibition of SIRT3 suppressed titanium particle-induced tumor necrosis factor-alpha (TNF-α), interleukin-1β (IL-1β) and interleukin-6 (IL-6) expression and prevented titanium particle-induced osteolysis and bone loss *in vivo*. This inhibition of osteoclasts differentiation was found to be linked to the downregulation and reduced phosphorylation of JNK and ERK. Taken together, inhibition of SIRT3 may be a potential target for titanium particle-induced bone loss.

## Introduction

Lower extremity total joint replacement surgery is associated with very positive outcomes, including a >90% average 15-20 year survival rate 1, 2. However, as rates of joint replacement surgery continue to rise, so too will rates of joint revision surgeries 3, 4. A number of causes may contribute to the need for revision, with aseptic loosening accounting for 55% and 31% of hip and knee revisions, respectively 5. While the mechanistic basis for aseptic loosening remains to be clarified, wear particle-induced peri-prosthesis osteolysis is thought to be an important driver of this process 6. Macrophages can take up wear particles, which subsequently stimulate them to produce a range of inflammatory factors including tumor necrosis factor-α (TNF-α), interleukin-1 β (IL-1 β), and interleukin-6 (IL-6), near the implant, thereby driving osteoclasts activation and pathological bone absorption 7. Excessive differentiation of osteoclasts is also closely related to other serious diseases, such as rheumatoid arthritis and osteoporosis 8, 9. Identifying novel molecular targets that can inhibit osteoclasts activation and differentiation may thus be an effective approach to treating of preventing bone loss-related diseases.

Osteoclasts are large multinuclear cells that form following macrophage/monocyte fusion and that facilitate bone resorption 10. Osteoclasts precursor proliferation and differentiation can be driven by macrophage phage colony-stimulating factor (M-CSF) and nuclear factor-κB ligand (RANKL), ultimately inducing the proliferation, migration, and fusion of these cells and thereby inducing osteoclasts development 11, 12. RANKL is a transmembrane protein from the tumor necrosis factor (TNF) superfamily, which plays an important role in osteoclasts formation. When RANKL binds to its cognate receptor, RANK, the adaptor protein TNF receptor-associated factor 6 (TRAF6) is recruited 13, 14. TRAF6, in turn, triggers a range of downstream MAPK and nuclear factor-κB (NF-κB) signaling pathways, ultimately resulting in nuclear NFATc1 activation, which is essential for osteoclasts differentiation and development 15-18. A number of anti-osteoclastsic compounds have been described to date, including bisphosphonates 19, calcitonin 20, and denosumab 21. These agents, however, exhibit significant side effects that hamper their clinical utility. For example, bisphosphonates can cause gastrointestinal symptoms, hypocalcemia, secondary hyperparathyroidism, and musculoskeletal pain 19. It is thus important that novel approaches to regulating osteoclasts development and activation be developed that are both safe and efficacious.

Sirtuin family members are NAD^+^-dependent deacetylases that control diverse metabolic processes in cells and thereby function as essential regulators of survival, autophagy, growth, DNA repair and duplication, oxidative stress, and mitochondrial biogenesis 22-25. There are seven known sirtuins (SIRT1-7), of which SIRT3 is known to be particularly important in the context of aging-related diseases such as cancer, cardiovascular disease, hearing loss, type 2 diabetes, and obesity 26. Bone marrow metabolism and systemic metabolism are closely linked to one another 27, 28, but the role of SIRT3 in osteolysis is not clear, and further study of how SIRT3 regulates bone metabolism is thus essential. The present study was designed to assess the importance of SIRT3 as a regulator of osteolysis and to explore any mechanisms underlying its role in this process.

Herein, we demonstrated that the inhibition of SIRT3 suppressed the development of osteoclasts in response to RANKL stimulation and reduced the production of pro-inflammatory cytokines. Furthermore, SIRT3 inhibition reduced titanium particle-induced osteolysis *in vivo*, indicating that SIRT3 may serve as an important regulator of bone metabolism.

## Materials and Methods

### Drugs and Reagents

3-(1H-1,2,3-triazol-4-yl) pyridine (3-TYP) (purity ≥99%) was obtained from Selleck (Shanghai, China) and dissolved in DMSO. PD184352 (CI-1040) was obtained from Selleck (Shanghai, China). Titanium (Ti) particles were purchased from Johnson Matthey Chemicals (MA, USA). Alpha modification of Eagle's medium (α-MEM), penicillin/streptomycin, and fetal bovine serum (FBS) were from HyClone (UT, USA). RANKL and M-CSF were from R&D Systems (MN, USA). Primary antibodies specific for phospho-NF-kB p65 (#3033), phospho-IkBα (#2859), NF-kB p65 (#8242), IkBα (#4814), phospho-ERK (#4370), phospho-p38 (#4511), phospho-JNK (#4668), ERK (#4695), p38 (#8690), and JNK (#9252) were from Cell Signaling Technology (MA, USA). MMP9 (ab76003) and NFATc1 (ab25916), CTSK (ab178647), c-fos (ab208942) were from Abcam (Cambridge, UK). Anti-rabbit and anti-mouse HRP-conjugated secondary antibodies were from Multi Sciences (Shanghai, China).

### Cell culture and osteoclasts differentiation

Primary bone marrow-derived macrophages (BMMs) were obtained using bone marrow isolated from the femurs and tibiae of 6-week-old female C57BL/6 mice. These cells were cultured in α-MEM containing 10% FBS, 1% penicillin/streptomycin, and 30 ng/mL M-CSF in a 37 °C, 5% CO2 incubator until 90% confluent. Non-adherent cells were discarded after 24 h, while the remaining adherent cells were cultured for an additional 3 days. BMMs were then plated at 8×10^4^/well in 24-well plates (Corning, NY, USA), after which they were stimulated with RANKL (50 ng/mL) and M-CSF (30 ng/mL). Following a 6 day culture period, TRAP staining was conducted, and cells with 3+ nuclei are considered mature osteoclasts.

### Cytotoxicity assay

A CCK-8 assay kit (ApexBio, MA, USA) was used to assess the cytotoxic impact of 3-TYP on BMMs. Briefly, these cells were added to 96-well plates until adherent, at which time they were treated for 48 h with a range of 3-TYP concentrations. Next, 10 μL of CCK-8 buffer was added per well for 2 h during which cells were incubated at 37 °C. Absorbance at 450 nm in each well was then assessed via microplate reader (BioTek, VT, USA).

### TRAP staining

BMMs were plated at 8×10^4^/well in 24-well plates, after which they were stimulated with RANKL (50 ng/mL) and M-CSF (30 ng/mL) in the presence of a range of 3-TYP concentrations (0, 25, 50 and 100 μM) to induce osteoclasts differentiation. The differentiation of these cells was then assessed via microscopy and TRAP staining conducted using a TRAP staining kit (Sigma-Aldrich, MO, USA). TRAP-positive cells with 3+ nuclei were considered to be osteoclasts and these cells were counted using the Image J software (NIH, MD, USA).

### SIRT3 knockdown by siRNA

To silence the SIRT3 gene, a siRNA targeting SIRT3 was designed and purchased from geneparma (Suzhou, China). Then special SIRT3 siRNA or non-binding control siRNA was transfected into BMMs using Lipofectamine RNAiMax reagent (Invitrogen, Carlsbad, CA, USA) according to the manufacturer's instructions. Briefly, BMMs were cultured in 6-well plates (3×10^4^/well) in α-MEM medium before transfection, After 6 h, the medium was replaced with α-MEM containing 10% FBS. 48 h later, the efficiency of transfection was observed under fluorescence microscope. The sequence of SIRT3 siRNA oligonucleotide as follow: 5-GACCUUUGUAACAGCUACATT-3, the antisense is 5-UGUAGCUGUUACAAAGGUCTT -3.The sense of negative control siRNA is 5- UUCUCCGAACGUGUCACGUTT-3, the antisense is 5-ACGUGACACGUUCGGAGAATT-3.

### Podosome belt staining

BMMs were plated at 8×10^4^/well in 24-well plates, after which they were stimulated for 5 days with RANKL (50 ng/mL) and M-CSF (30 ng/mL) in the presence of a range of 3-TYP (0, 25, 50 and 100 μM). Cells were then stained using DAPI and phalloidin, after which F-actin and cellular nuclei were imaged via fluorescence microscopy (Zeiss, Dresden, Germany).

### Resorption pit assay

Resorption pit assays were conducted to assess osteoclasts functionality. Briefly, BMMs were plated at 8×10^4^/well in Osteo Assay Surface 24-well plates (Corning, USA)^29^ , after which they were stimulated for 7 days with RANKL (50 ng/mL) and M-CSF (30 ng/mL) in the presence of a range of 3-TYP (0, 25, 50, 100 μM). After that, the cells were trypsinized and washed 3 times with PBS. The images were captured using an ordinary optical microscope (Zeiss), and Image J (NIH, MD, USA) was used to analyze the resorption area.

### RT-PCR

BMMs were cultured at 1×10^5^ cells/well in 6-well plates in α-MEM containing RANKL (50 ng/mL) and M-CSF (30 ng/mL). At appropriate time points, Trizol (Beyond, Shanghai, China) was used to extract total cellular RNA, and cDNA was then synthesized. Next, qRT-PCR reactions were conducted using a 10 μL reaction volume containing 5 μL SYBR Green QPCR Master Mix (Yeasen, Shanghai, China), 3 μl RNase-free H_2_O (Abcam, Cambridge, UK), 1 μl cDNA, and 0.5 μL each of the forward and reverse primers. Thermocycler settings were as follows: 95 °C for 10 minutes; 40 cycles of 95 °C for 10 s, 60 °C for 20 s, and 72 °C for 90 s. GAPDH served as a normalization control, and primer sequences are shown in Table S1.

### Immunofluorescent staining

Immunofluorescent staining for NFATc1 and MMP9. MMP9 and NFATc1 were detected via immunofluorescent staining. Briefly, cells were stained overnight at 4 °C with antibodies specific for MMP9 and NFATc1 (1:250; Abcam). Cells were then rinsed for 5 minutes with PBS, after which they were stained for 1 h with Goat Anti-Rabbit IgG H&L (Alexa Fluor 647) at 37 °C. Phalloidin and DAPI were then used to counterstain the cytoskeleton and nuclei of these cells, and a fluorescence microscope (Zeiss) was used for cellular imaging.

### Western blot analysis

BMMs were cultured at 6×10^5^ cells/well in 6-well plates, and were cultured in the presence of RANKL (50 ng/mL), M-CSF (30 ng/mL), and a range of 3-TYP concentrations. Cells were then lysed with RIPA buffer, after which a BCA assay kit (Beyotime Biotechnology, Shanghai, China) was used to quantify protein levels in isolated samples. Protein was then separated via SDS-PAGE and transferred to nitrocellulose membranes (Beyotime) that were blocked for 1 h with QuickBlock™ blocking buffer (Beyotime). Blots were next probed overnight with primary antibodies at 4 °C, and were washed thrice with TBST (10 minutes/wash) followed by a 1 h incubation with an appropriate secondary antibody. Protein bands were then detected via enhanced chemiluminescence (Sigma-Aldrich, MO, USA).

### Murine model of Ti particle-induced calvarial osteolysis

The Institutional Animal Ethics Committee of the First Hospital of Soochow University approved all animal experiments described herein. Briefly, 40 female C57BL/6 mice (6-8 weeks old) were randomized into four treatment groups (n=10/group): (1) a sham group (PBS only), (2) a vehicle group, (3) a low-dose 3-TYP group (25 mg/kg), and (4) a high-dose 3-TYP group (50 mg/kg). All groups other than the sham group underwent a 20 mg Ti particle-stimulation procedure. Briefly, after anesthesia, make an 6 mm sagittal incision in the middle of the skull, 20 mg Ti particles were evenly embedded under the periosteum on the surface of the parietal bone, and then the incision was sutured. Whereas in the sham group, the incision was closed immediately with no further intervention. All mice were intraperitoneally injected with the appropriate solutions every other day for 2 weeks, after which animals were euthanized and skull caps were collected and fixed using 4% paraformaldehyde.

### Micro-CT imaging

Murine skull samples were first fixed for 24 h in 10% neutral formalin, after which a Skyscan 1176 micro-CT (Aartselaar, Belgium) was used to conduct micro-computed tomography (micro-CT) scans of these samples (n = 5/group) with the following settings: 9 μm equidistant resolution, 50 kV, and 200 μA energy. Three-dimensional reconstructions were conducted using X-ray CT analyzer (SkyScan), which was also used for optical measurements of assorted tissue morphology parameters including the bone volume/tissue volume (BV/TV), bone mineral density (BMD), number of pores, and percentage of porosity.

### Histological analysis

After collection, samples were decalcified for 4 weeks in a 10% EDTA solution at 37 °C, after which they were paraffin-embedded, and sections were prepared with an appropriate instrument (Leica 2135, Germany). These sections were subjected to TRAP and hematoxylin and eosin (H&E) staining, after which an axiovert 40C optical microscope (Zeiss, Germany) was used for sample analysis.

### Statistical analysis

Data are given as means ± standard deviation (SD), and were analyzed using GraphPad Prism 8.0 (GraphPad Software Inc. CA, USA). Data were compared between groups via ANOVAs with Tukey-Kramer's post-hoc test. *p< 0.05, **p < 0.01, *** p < 0.005.

## Results

### Effect of SIRT3 on the development of RANKL-induced osteoclasts *in vitro*

**Figures 1:**
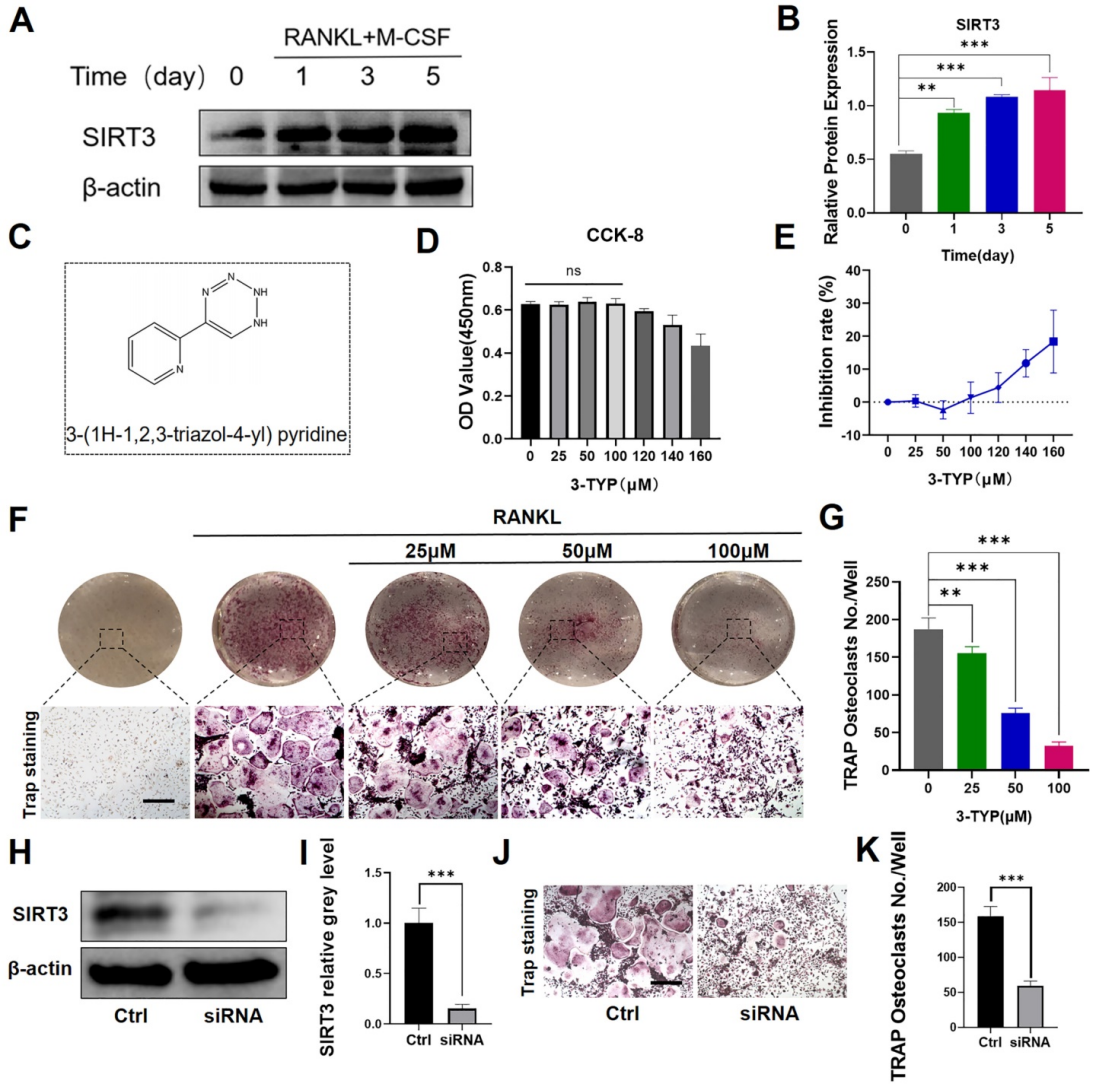
** Effect of SIRT3 on the development of RANKL-induced osteoclasts *in vitro*.** (A) SIRT3 expression was assessed via Western blotting at the indicated time points. (B) Results of western blotting are quantified. (C) 3-TYP structure and chemical formula. (D) Bone marrow macrophages (BMMs) were cultured in α-MEM containing RANKL, M-CSF and treated with different concentrations of 3-TYP, CCK-8 kit was used to detect cell proliferation. (E) Inhibition rate of 3-TYP on BMMs. (F) TRAP staining was used to analyze osteoclasts differentiation, revealing that 3-TYP inhibits osteoclastogenesis in a dose-dependent fashion. (G) TRAP-positive multinucleated cells and osteoclasts (nuclei ≥ 3) in each well were quantified. (F) BMMs were transfected with SIRT3 siRNA or scrambled siRNA (control). (I) Results of western blotting are quantified. (J) TRAP staining. (K) TRAP-positive multinucleated cells were quantified. Scale bar: 100 µm. Data are means ± SD (*p<0.05, **p<0.01, ***p<0.005, n= 3).

**Figures 2:**
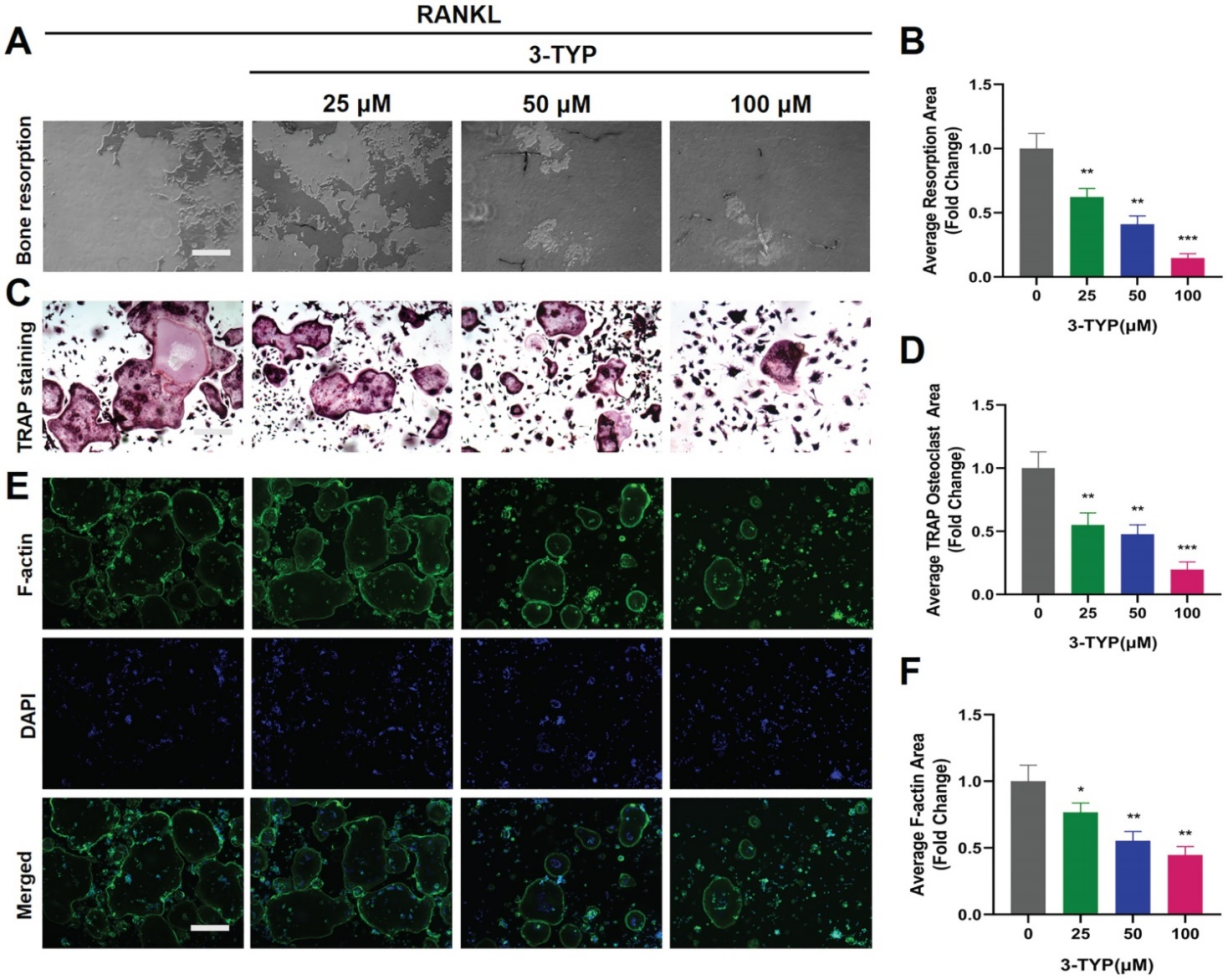
** Inhibition of SIRT3 suppresses F-actin ring formation and bone resorption.** (A) Resorption pits were assessed via inverted microscope. (B) Image J was used to measure pit area values. (C) TRAP staining was used to analyze osteoclasts differentiation (D) TRAP positive cells are quantified. (E) Osteoclasts were stained for F-actin (green) and nuclei (blue). (F) Quantification of the F-actin ring number per well. Scale bar: 100 µm. Data are means ± SD (* p<0.05, ** p<0.01, ***p<0.005, n=3).

We firstly found that SIRT3 expression was increased significantly in RANKL-induced osteoclasts relative to levels in untreated BMMs (Fig. 1A-B). SIRT3 expression rose with osteoclasts activation, indicating that SIRT3 may play an important regulatory role in this process. We additionally began by assessing the ability of the selective SIRT3 inhibitor 3-TYP (Fig. 1C) to induce direct cytotoxicity in murine BMMs over a 48 h culture period *in vitro,* revealing that doses under 100 μM had no impact on cell viability (Fig. 2D-E). We also demonstrated that 3-TYP suppressed SIRT3 expression during RANKL-induced osteoclasts formation (Fig. S1). We then explored the ability of 3-TYP to influence osteoclasts differentiation by treating BMMs with RANKL (50 ng/mL), M-CSF (30 ng/mL), and a range of 3-TYP concentrations, revealing that 3-TYP inhibits osteoclasts formation in a concentration-dependent manner (Fig. 1F-G). To more fully understand how 3-TYP inhibits osteoclastsogenesis, BMMs were then treated with 100 μM 3-TYP for different time intervals during the differentiation process (0‐2, 2‐4, 4‐6, and 0‐6 days). While 3-TYP treatment from days 0-2 dramatically inhibited osteoclasts differentiation, treatment from days 4-6 did not affect this differentiation process, suggesting that 3-TYP functions to suppress the early stages of RANKL-induced osteoclasts differentiation without causing cytotoxicity (Fig. S2). Finally, we further demonstrate the SIRT3-dependent function involved in the formation of osteoclasts, SIRT3 siRNA was employed to knock down its endogenous expression and determine the effects on RANKL-induced osteoclastogenesis. Western blotting confirmed that SIRT3 siRNA could significantly reduce the endogenous expression of SIRT3 (Fig. 1H-I). In addition, SIRT3 siRNA reduces the number of osteoclasts, compared with treatment with control siRNA (Fig. 1J-K). Collectively, these results indicate that inhibition of SIRT3 inhibits RANKL-induced activation of osteoclasts.

### Inhibition of SIRT3 suppresses F-actin ring formation and bone resorption

We next utilized a pit formation assay as a means of gauging the impact of SIRT3 on bone resorption, revealing that 3-TYP reduced bone resorption area in a dose-dependent fashion (Fig. 2A-B). Marked reductions in bone resorption area were observed following sample pretreatment with 3-TYP, particularly at high doses (100 μM). At the same time, TRAP staining was used to record osteoclasts (Fig. 2C-D). Phalloidin was also used to stain these cells in order to assess F-actin ring formation as a metric for osteoclasts function 30, 31. This analysis indicated that 3-TYP pretreatment was sufficient to reduce F-actin ring formation (Fig. 2E-F), consistent with the bone resorption assay results.

### Inhibition of SIRT3 suppresses osteoclast-associated gene and/or protein expression

**Figures 3:**
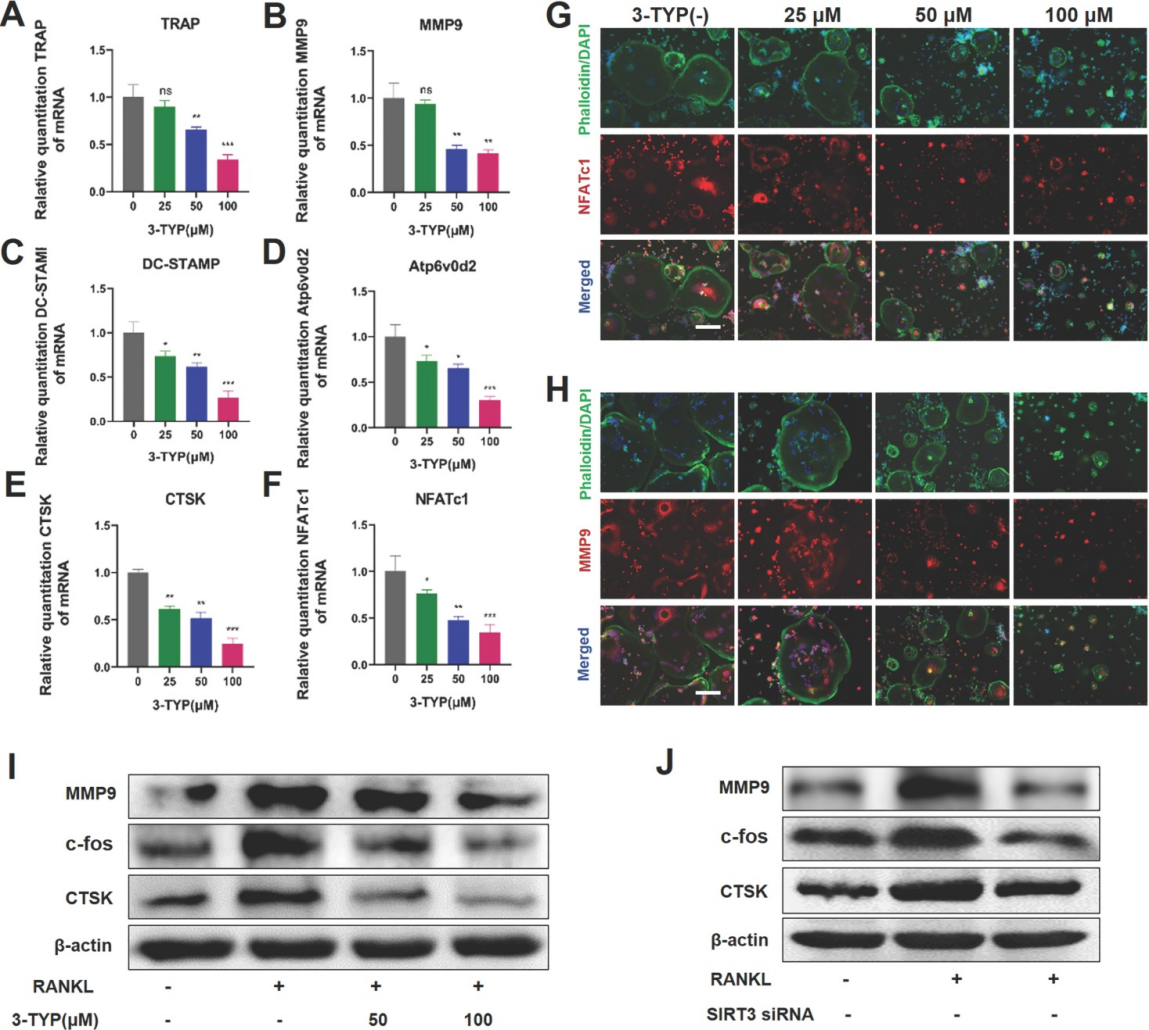
** Inhibition of SIRT3 suppresses RANKL-induced osteoclast- associated protein and/or gene expression.** (A-F) Expression osteoclast-associated genes (TRAP, MMP9, DC-STAMP, Atp6v0d2, CTSK and NFATc1) in BMMs following treatment for 24 h with a range of 3-TYP concentrations. (G-H) NFATc1 and MMP9 expression was assessed via immunofluorescent analyses. (I) MMP9, c-fos and CTSK expression was assessed via Western blotting under 3-TYP treatment. (J) MMP9, c-fos and CTSK protein expression under SIRT3 siRNA treatment. Scale bars: 50 µm. Data are means ± SD (* p <0.05, ** p <0.01, ***p <0.005, n=3).

To further assess the impact of SIRT3 on osteoclastsogenesis, we next analyzed the expression of key osteoclast-associated genes such as TRAP, MMP9, DC-STAMP, Atp6v0d2, CTSK and NFATc1, we found that 3-TYP suppressed the expression of these genes in a dose-dependent fashion (Fig. 3A-F). Immunofluorescent staining of the actin rings and NFATc1 or MMP9 shows that inhibition of SIRT3 reduces the size of osteoclasts and expression of NFATc1 and MMP-9 (Fig. 3G-H). Western blotting confirmed that the expressions of osteoclastic makers such as MMP-9, c-fos and CTSK are down regulated after exposure to 3-TYP and SIRT3 siRNA (Fig. 3I-J).

### Inhibition of SIRT3 prevents RANKL-induced MAPK signaling

**Figures 4:**
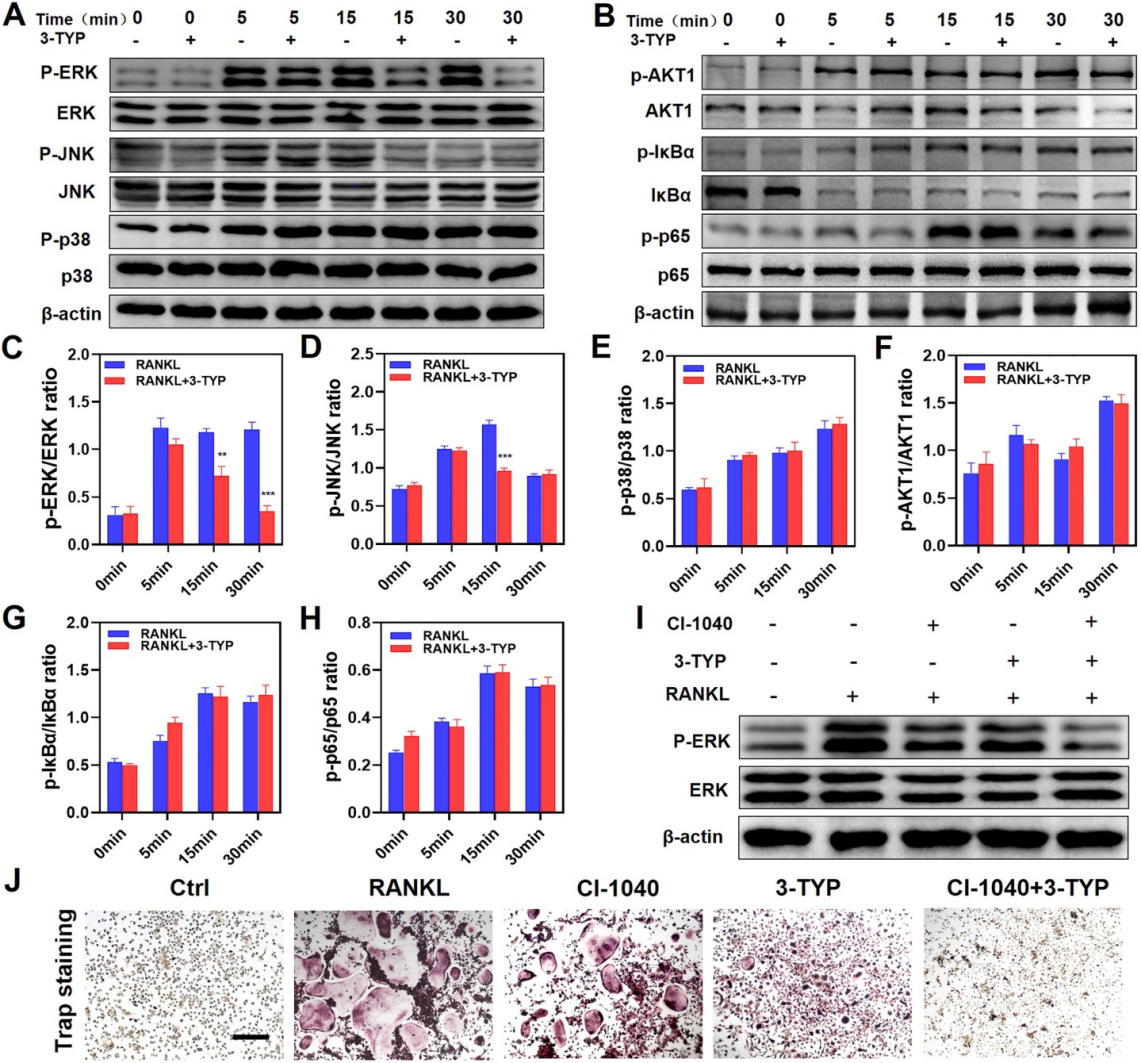
** Inhibition of SIRT3 inhibits RANKL-induced MAPK signaling.** (A) BMMs were treated for 12 h with 3-TYP, after which they were treated for 0-30 minutes with RANKL (50 ng/mL) and M-CSF (30 ng/mL). Western blotting was then used to measure ERK, JNK and p38 expression and phosphorylation. (C-E) Western blotting data were quantified respectively. (B) AKT1, IκBα and p65 expression and activation were assessed via Western blotting. (F-H) Western blotting data were quantified respectively. (I) ERK expression and phosphorylation at 15 minutes with RANKL (50 ng/mL) and M-CSF (30 ng/mL). (J) TRAP staining was used to analyze osteoclasts differentiation, revealing that ERK signaling play a significant role in osteoclastogenesis. Scale bars: 100 µm. Data are means ± SD (*p <0.05, **p<0.01, ***p<0.005, n=3).

To assess the mechanistic basis whereby inhibition of SIRT3 suppresses osteoclastic differentiation, we next analyzed the NF-κB, MAPK, and PI3K/AKT pathways in these cells via Western blotting given that these pathways are essential regulators of this differentiation process. With respect to MAPK signaling, 3-TYP pretreatment markedly inhibited both ERK and JNK phosphorylation without altering total protein levels (Fig. 4A). In contrast, 3-TYP did not significantly alter the phosphorylation of P65, IκBα, PI3K/AKT in these cells, suggesting that NF-κB and PI3K/AKT signaling were unaffected by this inhibitor (Fig. 4B). Figures 4C-H are the quantitative results of pathway-related proteins respectively. To further demonstrate that these changes in JNK and ERK signaling can account for the observed changes in osteoclastogenesis. We screened the ERK inhibitor PD184352 (CI-1040) to support the important role of ERK in osteoclasts activation. At 15 minutes, we found that CI-1040 inhibits the phosphorylation of ERK (Fig. 4I). Simultaneously, TRAP staining showed that CI-1040 significantly reduced TRAP-positive cells compared with the control, and the combination of CI-1040 and 3-TYP inhibited osteoclasts more obviously, which was attributed to the important role of the ERK signaling pathway in osteoclasts activation (Fig. 4J). Together, these data suggested that inhibition of SIRT3 suppresses RANKL-induced osteoclastsogenesis via disrupting MAPK signaling.

### Inhibition of SIRT3 dampens Ti particle-induced inflammatory cytokine production

**Figures 5:**
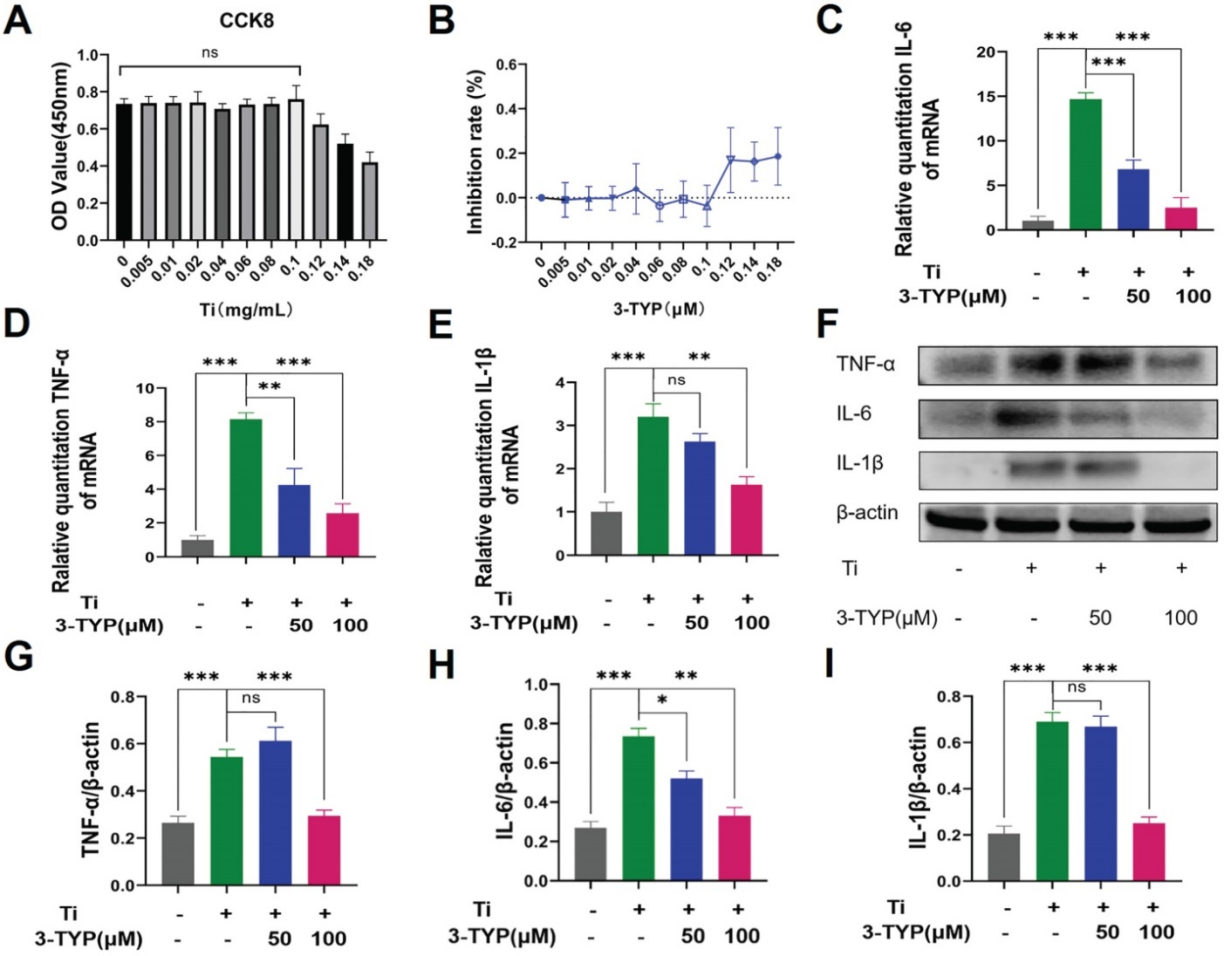
** Inhibition of SIRT3 suppresses inflammatory cytokine release.** (A) RAW 264.7 macrophages were cultured in different concentrations of the Ti particles for 72 hours. Cell viability was tested using cck-8. (B) Inhibition rate of Ti particles on RAW 264.7 macrophages. (C-E) RAW 264.7 macrophages were treated for 12 h with a range of 3-TYP doses, after which they were combined with Ti particles (0.1mg/mL). The expression of pro-inflammatory cytokine genes (IL-6, TNF-α and IL-1β) was then assessed via RT-PCR. (F) Following a 24 h pretreatment with the indicated 3-TYP concentrations, RAW 264.7 macrophages were treated with Ti particles (0.1 mg/mL), and inflammatory cytokine levels (TNF-α, IL-6 and L-1β) were assessed via Western blotting. (G - I) Western blotting data were quantified. Data are means ±SD (*p<0.05, **p<0.01, ***p<0.005, n=3).

Pro-inflammatory cytokines are important mediators of osteoclasts development and survival 32, 33, and macrophages are the primary producers of wear debris-induced pro-inflammatory cytokine production in the context of osteolysis. Above all, we evaluated the cytotoxicity of different concentrations of titanium particles on RAW264.7 macrophages, and revealed that doses below 0.1 mg/L have no effect on cell viability (Fig. 5A). The inhibition rate is shown in the Figure 5B. As such, we next stimulated RAW264.7 macrophages with Ti particles (0.1 mg/mL) and a range of 3-TYP concentrations and explored consequent cytokine production. We found that Ti particles induced significant increases in levels of inflammatory factors including IL-6, TNF-α and IL-1β (Fig. 5C-E), whereas 3-TYP treatment inhibited the production of all of these cytokines at the RNA and protein levels (Fig. 5F). Protein quantification is shown in Figure 5G-I. This suggested that 3-TYP functions as an inhibitor of Ti-induced inflammatory activity.

### Inhibition of SIRT3 prevents Ti particle-induced osteolysis and bone loss *in vivo*

**Figures 6:**
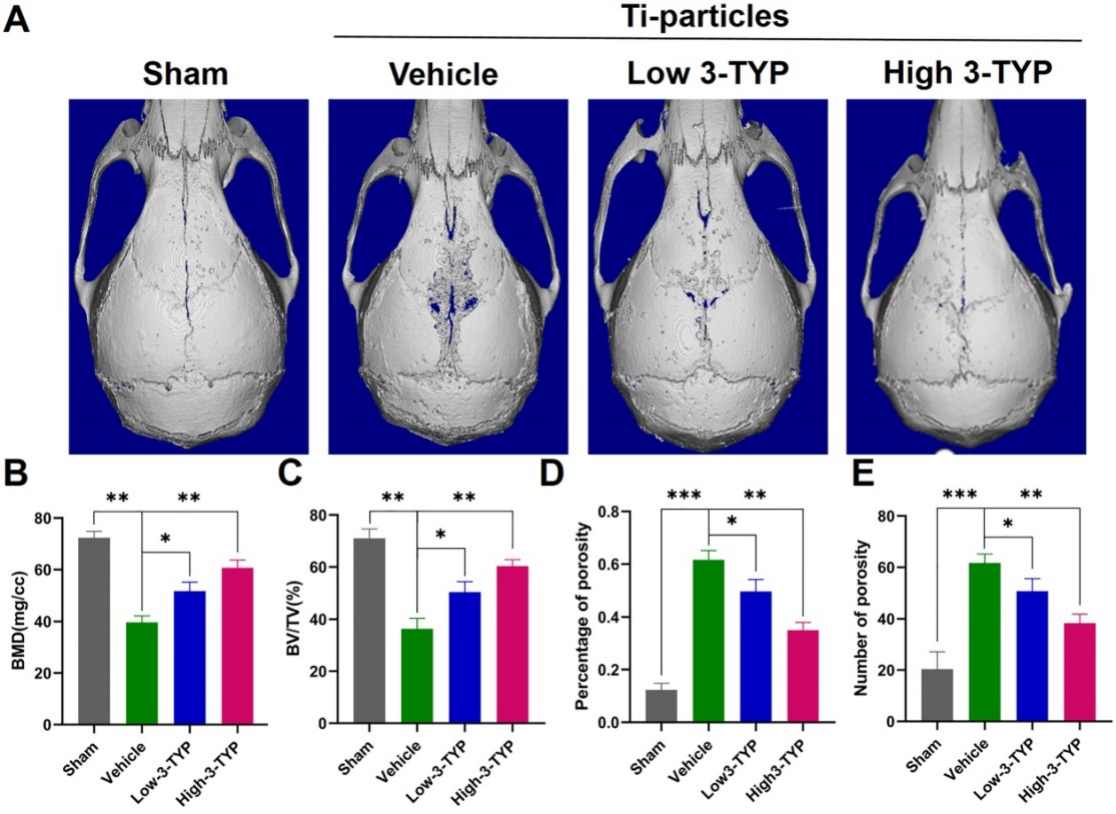
** Inhibition of SIRT3 prevents Ti particle-induced osteolysis and bone loss *in vivo*.** (A) Micro-CT analyses of calvaria samples from the indicated treatment groups. (B) Bone mineral density (BMD) values, (C) bone volume/tissue volume (BV/TV) values, (D) percentage of porosity and (E) number of porosity were quantified to assess bone microstructure. Data are means ± SD (*p <0.05, **p <0.01, *** p <0.005, n=5).

Given the promising efficacy of inhibition of SIRT3 as an inhibitor of osteoclastsogenesis and inflammation *in vitro,* we next assessed its efficacy *in vivo* by establishing a Ti particle-induced murine calvaria osteolysis model 34, which is commonly utilized to analyze aseptic loosening. Particle-induced erosion resulted in the formation of large lacunae (Fig. 6A), whereas 3-TYP treatment markedly alleviated this destruction. Indeed, bone parameter analyses revealed that BMD was significantly decreased in the vehicle group relative to the sham control group, whereas 3-TYP treatment prevented this reduction, particularly in animals administered a high 3-TYP dose (Fig. 6B). Mice that had been treated with 3-TYP also exhibited an increased bone volume/tissue volume (BV/TV) relative to the vehicle group (Fig. 6C), and exhibited fewer percentage of porosity and reduced number of porosity (Fig. 6D-E).

**Figures 7:**
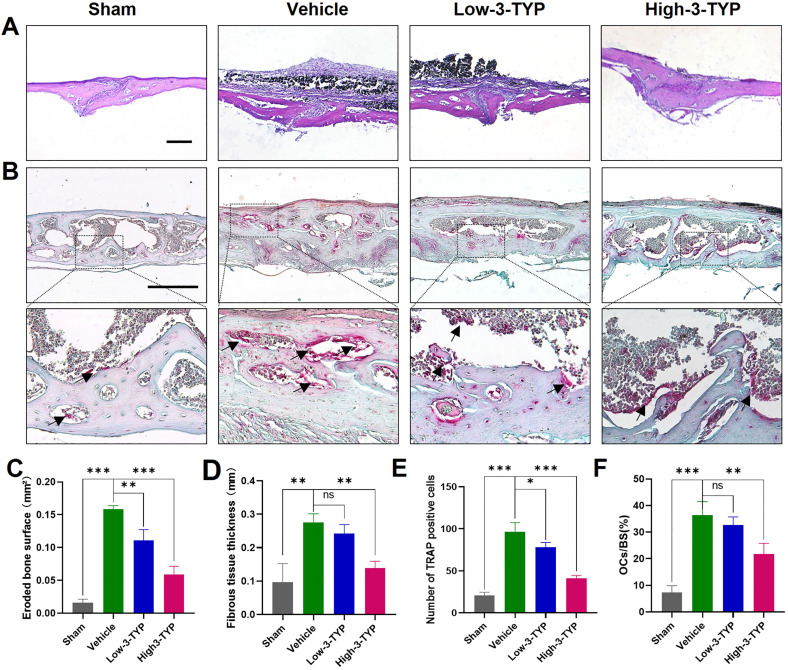
** Inhibition of SIRT3 inhibits Ti particle-induced bone loss and reduced osteoclasts number.** (A-B) H&E and TRAP staining were conducted. (C) Eroded bone surface, (D) Fibrous tissue thickness, (E) numbers of TRAP-positive cells, and (F) percentage of osteoclasts per bone surface (OCs/BS, %) were determined. Scale bar: 200 µm. Data are means ± SD (*p<0.05, **p<0.01, ***p<0.005, n=5).

Further histological analyses confirmed that inhibition of SIRT3 was able to prevent Ti particle-induced osteolysis (Fig. 7A). Analyses of H&E-stained sections indicated that the erosion surface area had increased by nine-fold in the vehicle group relative to the sham-operated control group, and fibrous tissue thickness had increased by seven-fold in vehicle group samples. Importantly, 3-TYP treatment dramatically reduced the erosion surface and fibrous tissue area relative to that observed in vehicle group animals (Fig. 7C-D). TRAP staining results revealed that relative to vehicle group animals, numbers of TRAP-positive cells in the 3-TYP treatment group were significantly reduced (Fig. 7E), as was percentage of osteoclasts per bone surface (OCs/BS, %) (Fig. 7F).

## Discussion

Peri-prosthetic osteolysis and associated aseptic loosening are the most common long-term complications of joint replacement surgery, increasing the complexity of arthroplasty procedures 35. While the mechanistic basis for such osteolysis remains incompletely understood, osteoclast-mediated bone resorption is known to be a primary driver of this process. Inhibiting osteoclasts activation and associated inflammation thus represent viable approaches to preventing or treating wear debris-induced peri-prosthetic osteolysis. In the present study, we determined that activated osteoclasts upregulate SIRT3, suggesting it may be involved in their differentiation and/or activation. We therefore treated osteoclasts with the SIRT3-selective 3-TYP inhibitor. Concentrations of 3-TYP that were under 100 μM did not adversely affect BMMs proliferation or survival, whereas in bone resorption assays we found that 3-TYP suppressed osteoclasts function in a dose-dependent fashion. Importantly, H&E staining and micro-CT analyses showed that 3-TYP was sufficient to prevent bone loss *in vivo* in a Ti particle-induced osteolysis model system. We also found that 3-TYP treatment reduced inflammatory cytokine production, which is noteworthy owing to the pathogenic role of these molecules in the context of osteolysis. In summary, these results indicated that inhibition of SIRT3 plays a key role in suppressing osteoclasts activation and inflammatory cytokine production, thereby suppressing Ti particle-induced bone loss.

We additionally assessed the mechanisms whereby 3-TYP inhibits osteoclasts differentiation and activation. The MAPK, NF-κB and PI3K/AKT signaling pathways are the primary pathways that govern osteoclastsogenesis and subsequent osteoclasts maturation, and these pathways are also closely linked to pro-inflammatory cytokine signaling 35-38. The signaling activity of MAPK proteins including ERK, JNK, and p38 is essential to the differentiation and activation of normal osteoclasts 37. At a functional level, ERK activation can enhance the expression of the collagenase MMP-9, which can in turn facilitate osteoclasts migration and bone resorption 39. RANKL-induced activation of JNK leads to the phosphorylation of c-Jun, which complexes with c-Fos to yield an active transcription factor that is essential for osteoclastsic differentiation 40. Herein, we found that inhibition of SIRT3 was sufficient to inhibit RANKL-induced MAPK signaling via suppressing ERK and JNK activation, whereas did not affect NF-κB and PI3K/AKT pathway signaling.

SIRT3 functions as a deacetylase responsible for the majority of mitochondrial lysine acetylation 41, 42, and it has been linked to functional roles in a range of pathogenic contexts such as cancer, obesity, type 2-diabetes, cardiovascular disease, and hearing loss. The role of SIRT3 in bone metabolism, however, has remained controversial. Kim et al. 43 who believed SIRT3 activates mitochondrial SOD activation through deacetylation, reduces ROS production, thereby reducing bone breakdown caused by osteoclasts activation. Huh et al. 44 found that SIRT3-/- mice had increased osteoclasts. However, Ho et al. 45 in contrast found that SIRT3 was a positive regulator of adipogenesis and osteoclastogenesis and a negative regulator of skeletal homeostasis. Our research is consistent with Ho et al. who believe that SIRT3 plays a positive role in osteoclasts activation. Moreover, Inhibition of SIRT3 prevents the pathological bone destruction by reducing the excessive activation of osteoclasts in the Ti particle-induced mouse osteolysis model. Currently, we are not sure what caused the difference, it may be related to the process of Ti particle-induced osteolysis, which involves macrophages engulfing titanium particles and inducing inflammatory cytokine mainly include TNF-α, IL-1β and IL-6, these inflammatory factors play an important role in driving osteoclasts activation and pathological bone resorption 33.

Bone homeostasis is maintained by a balance between osteoclasts and osteoblasts activation *in vivo,* and disruptions in this homeostasis can result in serious metabolic bone disorders 46-48. Osteolysis can thus be effectively treated by mediating bone formation. Wear particles have been shown to impair the activation and viability of osteoblasts 49. Our data, however, do not rule out the possibility that inhibition of SIRT3 may impact osteoblastogenesis in addition to impacting osteoclasts differentiation. Indeed, Ding et al. 50 found SIRT3 to control mitochondrial function and to thereby serve as a key regulator of osteoblastic differentiation, indicating that overexpressing SIRT3 may thus drive bone formation via inducing osteoblasts development. As such, ongoing work in our laboratory seeks to resolve the impact of SIRT3 on osteoblasts differentiation in this experimental context.

There are multiple limitations to the present study. For one, our animal model is inconsistent with true peri-prosthetic osteolysis in human patients, as in humans this process occurs over extended periods of time due to the gradual accumulation of wear particles, unlike in our experimental model. Secondly, we only assessed local histological alterations within the skull and did not assess these animals for potential systemic side effects. Peri-prosthetic osteolysis is a complex process that requires cross-talk and cooperation among multiple cell types including osteoclasts and osteoblasts. Future research regarding the role of SIRT3 in osteoblasts is thus warranted.

In summary, our results confirmed that the inhibition of SIRT3 can decrease Ti particle-induced bone resorption, suppress osteoclastsic differentiation, and decrease inflammatory cytokine production. SIRT3 may therefore represent a viable therapeutic target in the context of peri-prosthetic osteolysis or other diseases associated with inflammation and osteoclastsic differentiation.

## Supplementary Material

Supplementary figures and tables.

## Figures and Tables

**Figures 8:**
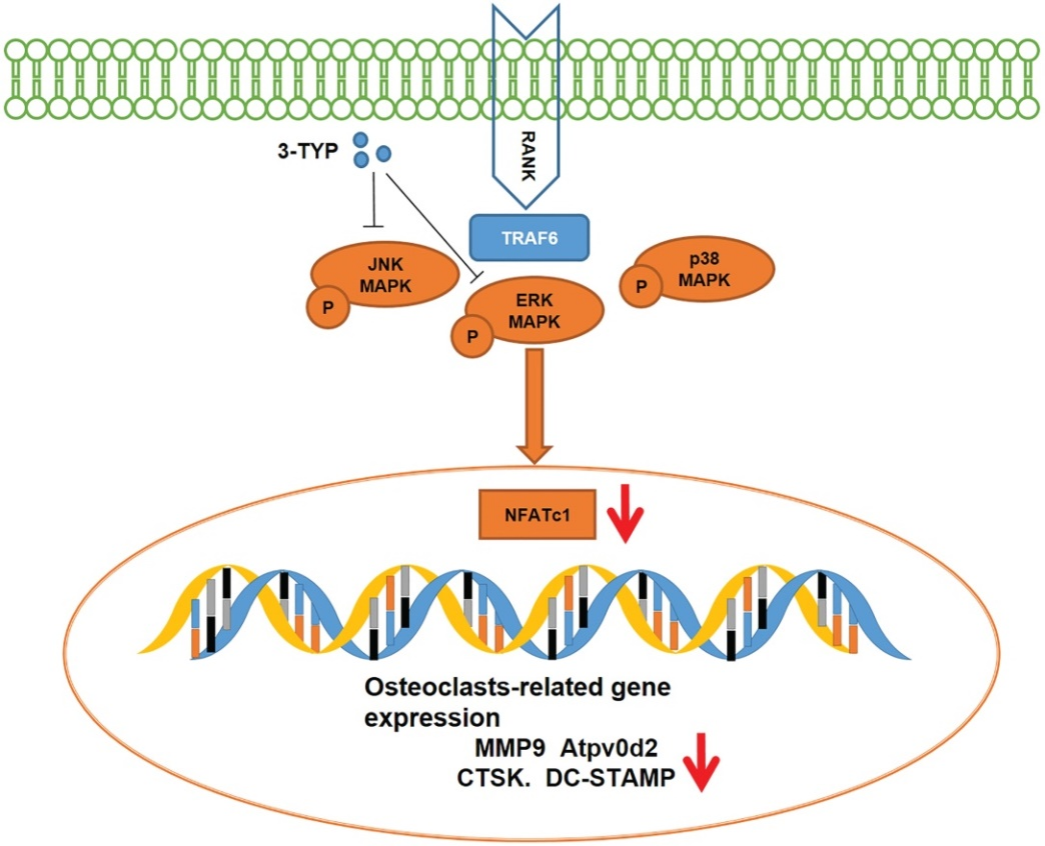
Schematic model of the signaling mechanism of 3-TYP inhibitory effect on Ti particle-induced skull osteolysis.
