# Supplementary material for: Inhibition of Sirtuin 3 prevents titanium particle-induced bone resorption and osteoclastsogenesis via suppressing ERK and JNK signaling
Source: Int J Biol Sci. 2021 Apr 3;17(5):1382–94. doi: 10.7150/ijbs.53992 (PMC8040473; doi:10.7150/ijbs.53992)
Supplement: Supplementary file 1 — Supplementary figures and tables. [file ijbsv17p1382s1.pdf]

Inhibition of Sirtuin 3 prevents titanium particle-induced bone resorption and osteoclastsogenesis via suppressing ERK and JNK signaling

Ning Li [1*], *Xiaoping Li* [1*], *Kai Zheng* [1*], *Jiaxiang Bai* [1], *Weicheng Zhang* [1], *Houyi Sun* [1], *Gaoran Ge*[1], *Wei Wang* [1], *Zhen Wang* [2], *Ye Gu*[3] *Yi ,Xue*[4], *Yaozeng Xu*[1✉], *Dechun Geng* [1✉], *Jun Zhou*[1✉]

1. Department of Orthopaedics, The First Affiliated Hospital of Soochow University, No. 188 Shizi Street, Suzhou, Jiangsu 215006, P. R. China
2. Department of Orthopaedics, Suzhou Kowloon Hospital Shanghai Jiao Tong University School of Medicine, Suzhou, Jiangsu 215006, China.
3. Department of Orthopedics, Soochow University Affiliated First People's, Hospital of Changshou City, Changshu, Jiangsu 215500,China.
4. Department of Orthopaedics, Changshu Hospital Affiliated to Nanjing University of Traditional Chinese Medicine, Suzhou, Jiangsu 215500, China

* These authors contributed equally to this work.

✉ **Corresponding authors**:

Prof. *Jun Zhou.* Email: zhou.jun.roy@hotmail.com;

Prof. *Dechun Geng.* Email: szgengdc@suda.edu.cn;

Prof. *Yaozeng Xu.* Email: xuyaozeng@163.com.

Genes	Forward (5'-3')	Reverse(5'-3')
CTSK	GGGAGAAAAACCTGAAGC	CGAGACTCAGGGGTCTTA
NFATc1	CCGTTGCTTCCAGAAAATAACA	TGTGGGATGTGAACTCGGAA
TRAP	TGTGGCCATCTTTATGCT	TTCGGGGTTTCTTTACTG
MMP9	CTGGACAGCCAGACACTAAAG	CTCGCGGCAAGTCTTCAGAG
Atp6v0d2	GTGAGACCTTGGAAGACCTGAA	TCGGGGACTCGTGTAAAGAG
DC-STAMP	AAAACCCTTGGGCTGTTCTT	AATCATGGACGACTCCTTGG
IL-1β	ACTCATTGTGGCTGTGGAGA	TGTCCGAGGCTCTACTTGTT
IL-6	TCGTGGAAATGAGAAAAGAGTG	ACATACTTGTTGCTACTACGTGA
TNF-α	CTGAGGTCAATCTGCCCAAGTAC	GAAACCTCAGTAACGAGACACTTC
GAPDH	AGCCATGTACGTAGCCATCC	CTCTCAGCAGTGGTGGTGAA

Table S1: *Sequences of the primers used for RT-PCR. CTSK : cathepsin K ; NFATc1:nuclear factor of activated T－cell cytoplasmic 1 ; TRAP: Tartrate resistant acid phosphatase ; MMP9： matrix metallopeptidase 9 ; Atp6v0d2： ATPase H+ Transporting V0 Subunit D2 ; DC-STAMP： dendritic cell-specific transmembrane protein ; IL-1β: interleukin-1β, IL-6: interleukin-6 ; TNF-α: tumor necrosis factor-α; GAPDH: glyceraldehyde-3-phosphate dehydrogenase.*

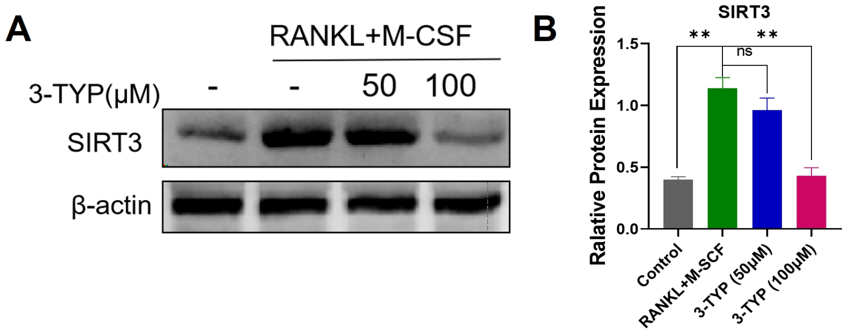

Figures S1. *3-TYP inhibits the increase of SIRT3 expression in the development of RANKL-induced osteoclasts. (A) SIRT3 expression was assessed via Western blotting at the day 5. (B) Results of western blotting are quantified. Data are means ± SD. (*p <0.05, **p<0.01, ***p<0.005, n=3).*

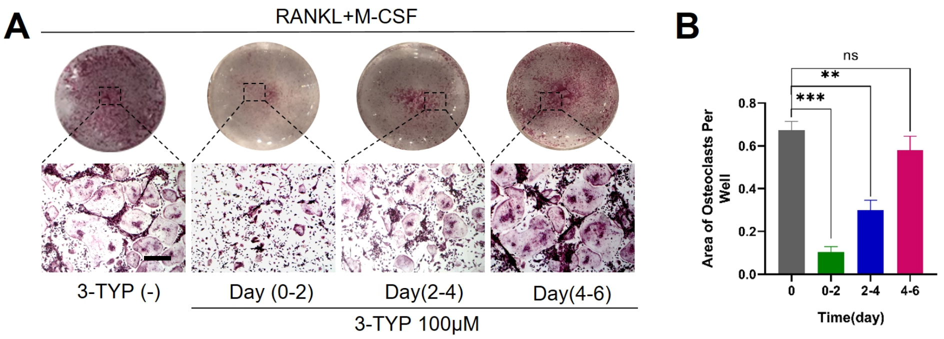

Figures S2. *3-TYP inhibits the RANKL-induced osteoclasts. (A) BMMs were cultured in media containing 100μM 3-TYP, RANKL (50ng/mL), and M-CSF (30ng/mL) for the indicated periods of time, revealing that 3-TYP suppresses osteoclasts differentiation during early time points. (B) Quantification of TRAP-positive multinucleated cells and osteoclasts per well (nuclei ≥ 3). Scale bar: 100μm. Data are means ± SD. (*p <0.05, **p<0.01, ***p<0.005, n=3).*

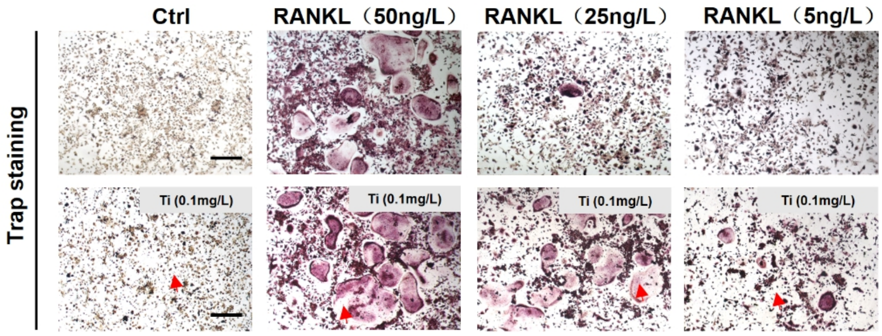

Figure S3. *Titanium particles promoted the differentiation of BMMs into mature osteoclasts in the presence of RANKL (Red arrow: titanium particles). Scale bar: 100μm.*

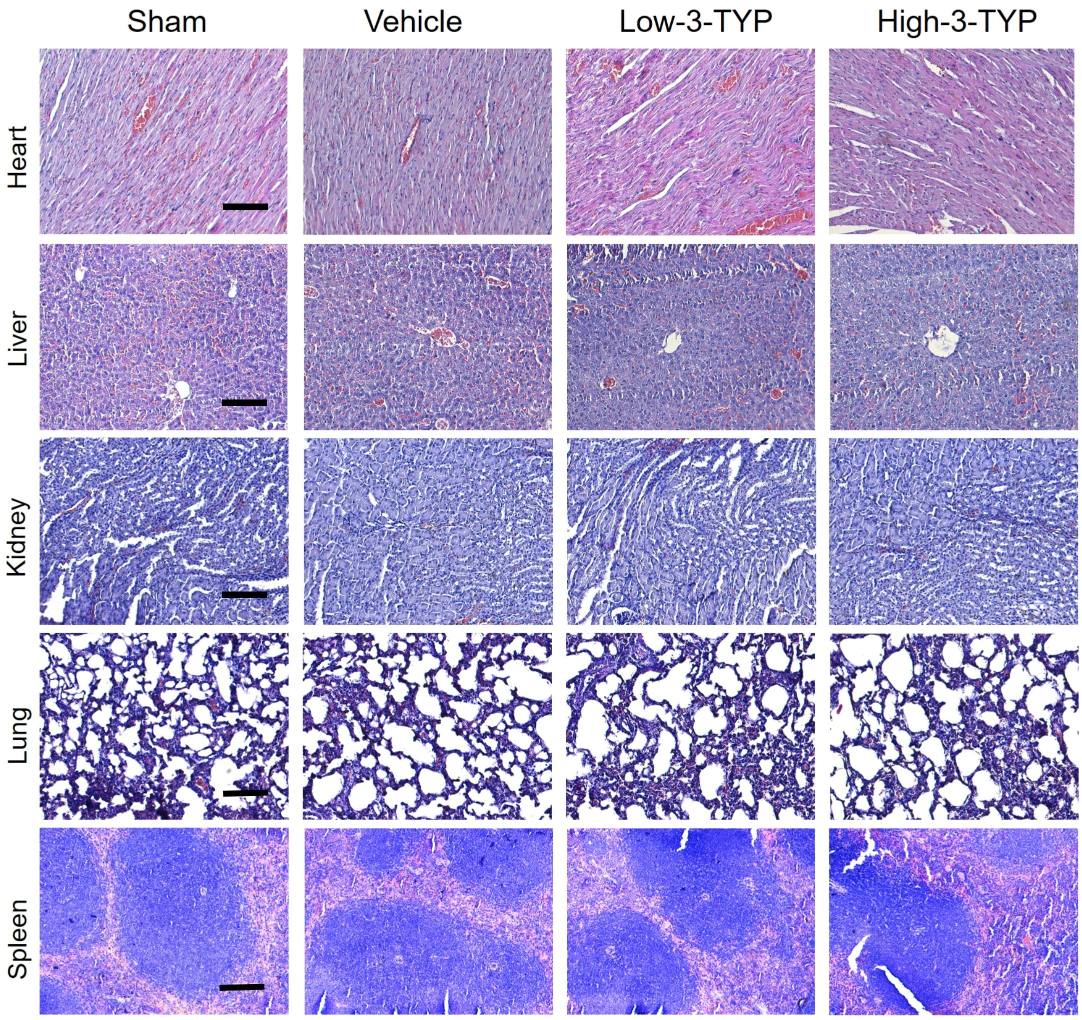

Figure S4. *Toxicities of 3-TYP on heart, liver, kidney, lung and spleen. H&E staining of the organ tissue sections. Scale bar: 50μm.*
